# Brownotate, a Comprehensive Solution to Generate Protein Sequence Databases for Any Species

**DOI:** 10.1002/pmic.70094

**Published:** 2026-01-06

**Authors:** Adrien Brown, Alexandre Burel, Sarah Cianférani, Christine Carapito, Fabrice Bertile

**Affiliations:** ^1^ Laboratoire de Spectrométrie de Masse Bioorganique (LSMBO) IPHC UMR7178 Université de Strasbourg, CNS Strasbourg France; ^2^ Infrastructure Nationale de Protéomique ProFI – FR2048 Strasbourg France

**Keywords:** genome annotation, genome assembly, pipeline, protein database, proteomics

## Abstract

Proteomics is strengthening research in biology and the diversification of the model organisms studied is very promising for fully understanding the complexity of biological principles. However, the lack of protein sequence databases for many species is a major bottleneck. Existing computational solutions are usually incomplete and/or only usable by bioinformaticians. We have built an open‐source, user‐friendly pipeline, called Brownotate, which allows anyone to generate protein sequence databases for any species as long as sequencing information is available. The pipeline can extract already existing protein sequences, but also automatically annotate any genome assembly or assemble and annotate any DNA sequence dataset. By testing the pipeline with numerous sequencing and assembly datasets covering a large part of the phylogenetic tree, we show that Brownotate generates fragmented but good quality assemblies and good quality annotations when compared to reference data. By comparing the use of protein databases generated by Brownotate or downloaded from NCBI to interpret proteomic data, we show very comparable results. The Brownotate pipeline is, therefore, an important new addition to the proteomics toolbox. The pipeline and its web interface are freely available at https://github.com/LSMBO/Brownotate and https://github.com/LSMBO/brownotate‐app, respectively.

**Summary:**

- This study evaluated the performance of a newly developed pipeline, Brownotate, for the assembly and annotation of sequencing data for multiple species, from prokaryotes to eukaryotes. We compared their fragmentation level (assembly) and completeness based on evolutionary expectations of gene content, and we evaluated their overlap. Brownotate generated fragmented, slightly less complete assemblies. However, the overlap of proteins predicted was very good, despite an excess of predicted sequences of small size with Brownotate. In addition, the interpretation of proteomics data downloaded from PRIDE repository for 27 species was found to lead to very similar results regardless of the origin of the protein sequencing database used, whether it was generated by Brownotate or downloaded from NCBI. Brownotate, made available to the community, will, therefore, be a tool of choice to mitigate the lack of an appropriate protein sequence database for many species, and allow proteomists to analyse without delay samples from species for which only sequencing data are available.

**Abbreviations**
BR: Brownotate‐generated datasets
OBRA: datasets generated using only Brownotate's annotation
REF: reference datasets

## 1 Introduction

Protein databases, gathering a wide range of protein‐related information, have many applications, including in comparative biology and biomedicine. In particular, protein sequence databases [1] are very useful resources for proteomics research, as they enable the identification of proteins from mass spectrometry (MS) data and link them to a series of structural and functional information [2, 3]. The repositories of the NCBI (e.g., SRA, GenBank, RefSeq) [4], EMBL‐EBI (Ensembl) [5] and Uniprot consortium (UniprotKB) [6] are among the main resources making sequencing data, genome assemblies and/or protein sequences publicly available to the community.

An appropriate protein sequence database is not always available for investigated organisms, thus hindering some innovative research [7]. This is obviously the case for species whose genome is not yet known or accessible, for which a database of protein sequences from a taxonomically close species needs to be used to analyse MS data, based mainly on conserved peptide sequences and the use of de novo sequencing [8]. However, this is not as efficient as having the protein sequences of the species under study. Proteogenomics, using customized protein sequence databases generated from genomic and transcriptomic sequence information, has proved to allow improving protein sequence databases [9] but this is not an easy task.

The lack of appropriate protein sequence databases is due to a major bottleneck in assembling and annotating sequenced genomes. In the NCBI databases (accessed March 2024), for example, sequencing data can be found for 122,329 eukaryote species, but 87% of these datasets have not been processed to generate an assembly, and 73% of available assemblies are not annotated. Scientists working on species whose genomes are not annotated must, therefore, wait for available assembly or annotation datasets to be curated and validated, or find a way to assemble and annotate the genomes themselves.

When a genome is sequenced, many DNA sequence reads are obtained, usually 50–50,000 bp long according to the platform used. To assemble the numerous DNA reads obtained, homology‐based assembly involves mapping to a reference genome from a model organism representative of the species under study [10]. Without a reference genome, de novo assembly works from scratch, relying on the manipulation of De Bruijn graphs, where overlaps between DNA reads are used to build longer contiguous sequences [10, 11, 12]. With an assembly, it becomes possible to annotate a genome, that is, to predict coding gene sequences through the recognition of structural features. Gene prediction uses either homology‐based or ab initio methods, or a combination of both [13, 14]. Homology‐based prediction relies on aligning the assembly under study with extrinsic DNA, RNA or protein sequences available in public databases, while ab initio prediction relies on computational algorithms based on Hidden Markov Models (HMMs) [15] to identify intrinsic gene features in DNA assembly sequences (start and stop codons, splicing sites).

To facilitate the sometimes difficult use of the many programs for assembling and annotating sequencing data, some of them have been grouped into pipelines [16, 17, 18, 19, 20, 21, 22, 23] (see also https://www.ncbi.nlm.nih.gov/refseq/annotation_euk/process/ and https://github.com/ncbi/rapt). These pipelines generally consider either only prokaryotic or only eukaryotic genomes and, to our knowledge, there is currently no widely recognized pipeline that performs both the assembly and annotation for eukaryotic species in a fully automated way. In addition, available pipelines do not automatically search all types of datasets available for a given species. Sometimes, there is a limitation in terms of the size of datasets that can be processed or the sequencing platform that can be considered. Other disadvantages include the fact that the choice of extrinsic sequences for homology‐based approaches is not always guided, the quality of assembled or annotated sequences is not always evaluated and a graphical interface is not always available.

Our aim was to develop a new, comprehensive, open‐source pipeline, to automate in a user‐friendly way for any non‐specialist the search and download of available datasets, as well as the assembly and annotation of both prokaryotic and eukaryotic genomes, in order to generate on‐demand protein sequence databases. With its excellent performance, Brownotate will make it easy to carry out proteomic analyses on an ever‐growing number of species.

## 2 Materials and Methods

### 2.1 The Brownotate Pipeline

**FIGURE 1:**
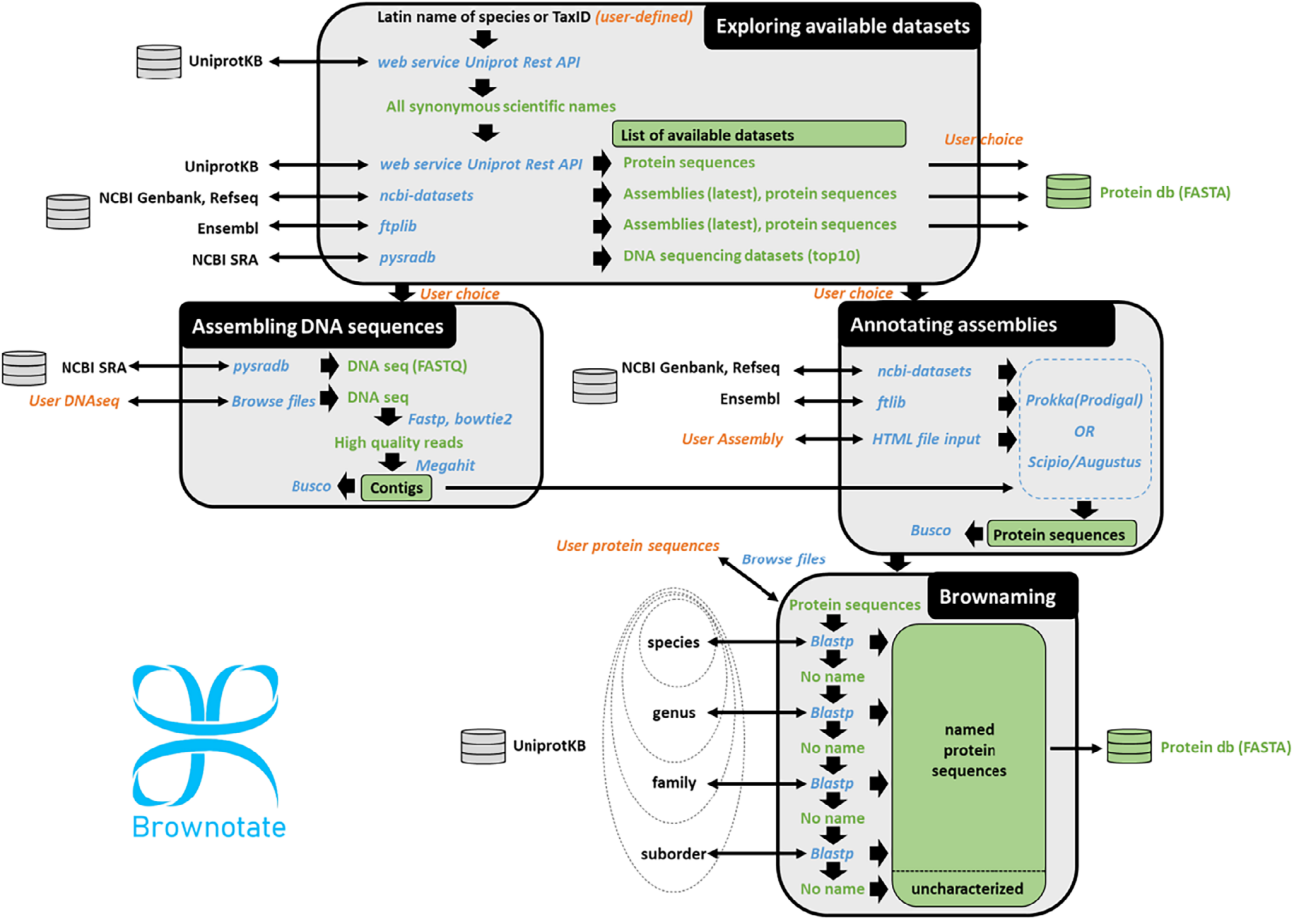
The Brownotate pipeline. The four modules of the Brownotate pipeline are shown with the user input in orange, the tools implemented in Brownotate in blue and the outputs in green.

A locally installed Brownotate pipeline brings together a series of open‐source programs, which enable different tasks to be performed sequentially (Figure 1).

### 2.2 Exploring Available Datasets

From a species Latin name or Taxonomy ID, all synonymous scientific names are retrieved from UniprotKB using the web service Uniprot Rest API. These names are then used to explore available datasets in the NCBI SRA database (DNA sequencing), Genbank, Refseq, ENSEMBL and UniprotKB databases (assemblies and/or protein sequences). Specifically, the NCBI SRA database is queried using the pysradb package [24], while prioritizing entries from short‐read platforms and WGS strategies and with an estimated sequencing depth between 50× and 100×. The NCBI (Genbank, Refseq) is queried using NCBI‐datasets tool [25], and the ENSEMBL database using ‘ftplib’, a Python package able to explore FTP servers and only the ‘latest’ datasets are retained. The UniprotKB databases are queried using the web service Uniprot Rest API to determine the number of protein entries in TrEMBL and Swiss‐Prot and whether a reference proteome is available. If no protein sequence datasets are found for the species under study, those available for phylogenetically close species are proposed. Users can decide to download an eventual protein database already available or to annotate an available assembly or assemble and annotate a sequencing dataset. To help users make this informed decision, retrieved datasets are directly linked (i.e., clickable) to the web page that describes them in their original database.

### 2.3 Assembling DNA Sequences

Using the SRAtoolkit v2.9.6 [26], NCBI DNA sequence datasets are downloaded in Fastq format [27]. Filtering is then performed using fastp v0.23.4 [28] to discard low‐quality DNA reads, trim low‐quality DNA read extremities and remove adapter sequences that had been useful during sequencing. For datasets originating from the Illumina platform, an additional step is performed using Bowtie2 v2.5.4 [29] to get rid of DNA reads that match the PhiX virus. The assembly of high‐quality reads is then performed using the MEGAHIT v1.2.9 algorithm [30]. To estimate the completeness of the assembly, Brownotate incorporates the tool Busco v5.7.1, which evaluates the proportion of universal single‐copy orthologs (hereafter referred to as Busco orthologs) found compared with all those expected for the species studied, according to its taxonomic class [31].

### 2.4 Annotating an Assembly

The prediction of coding sequences (CDS), that is, those that translate a gene into a protein, is more complex for eukaryotes than for prokaryotes, hence the use of different annotation tools.

For prokaryotes, we chose to use Prokka v1.14.6 [18], which is widespread and easy to use. Prokka uses one of the most powerful recognized tool [32], Prodigal [33], to identify sequences delimited by start and stop codons on the six reading frames. These candidate coding sequences then undergo validation (recognition of gene features) based on criteria such as length and GC content, and then translation into protein sequences that are exported as a FASTA file after completeness checking using Busco v5.7.1.

For eukaryotes, a combination of homology‐based and ab initio annotation methods is used. To gain insight into peculiar features (e.g., splicing sites, preferential codons for a given amino acid, frequent residues flanking genes) of the gene structure of the species under study, Brownotate uses Scipio 1.4.1, containing the BLAT alignment program [34], to compare the assembly of interest with the protein sequences of a taxonomically close species. The information thus obtained is then used by the Augustus 3.5.0 suite, one of the best program to train an HMM model [35], which predicts coding sequences and translates them into amino acid sequences [36]. Redundant sequences are then eliminated and the possibility is offered to users to discard very short sequences. Retained protein sequences are then exported as a FASTA file after completeness checking using Busco v5.7.1.

### 2.5 Propagating Protein Names

Brownotate is able to assign meaningful names to exported protein sequences using Brownaming (Figure 1), an in‐house developed tool that uses blastp v2.16.0 [37] to search UniprotKB for the most homologous protein known for the phylogenetically closest species. Protein names are hence propagated when the match is satisfactory (bitscore greater than or equal to 50). If no match or no satisfactory match is found, a second search is performed, extending the comparison to proteins from species at broader taxonomic levels, progressively widening the search space up to suborder level. All the information relating to the homologous entry used, including the phylogenetic proximity (shared taxonomic group and rank of the common ancestor), which reflects the degree of relationship between the two proteins and the details of the blast results, is stored in a table available to users among the output files given by Brownotate.

### 2.6 Brownotate Performance Evaluation

Brownotate's performance was assessed in terms of the quality of the assemblies and annotations obtained, but also by testing the use of the protein databases generated to analyse proteomics data. To do so, 75 available sequencing datasets (Table S1) for 44 different species distributed among different taxonomic groups, 44 assemblies (for 44 species) and 27 protein sequence datasets (for 27 species) (Table S2) were downloaded from NCBI databases. These assemblies and annotations are hereinafter referred to as “reference datasets” (REF).

### 2.7 Evaluating Assembly and Annotation Quality

The assemblies and annotations generated using Brownotate are hereafter referred to as “Brownotate‐generated (BR)” when starting from sequencing datasets, or “only Brownotate's annotation module (OBRA)” when starting from REF assemblies. Importantly, it should be reminded here that, to avoid bias and simulate a scenario in which we have no prior information on the species under study, proteins from a different organism have been used here as extrinsic sequences to train the HMM model to generate the BR and OBRA protein sequence databases, that is, excluding the use of REF annotations (Table S3).

For 44 different species, BR assemblies were compared to REF assemblies using Quast v5.0.0 [38], which calculates various key metrics, including total assembly length (cumulative length of all contigs), N50 (shortest contig length to be included to cover the first half of an assembly after all contigs have been sorted by decreasing size) and the proportion of the REF assembly covered by the BR assembly. In addition, the completeness of BR and REF assemblies was compared using Busco v5.7.1.

We also evaluated the influence of the type of genomic dataset selected as input, that is, depending on the sequencing platform (e.g., Illumina, Oxford Nanopore, IonTorrent, PacBio) or strategy (e.g., WGS, Mnase, CHiP, Hi‐C, PCR) used to generate them and on their sequencing depth. To this end, the same key metrics were used as presented above to compare Brownotate assembly of genomic datasets (i) from different sequencing platforms or strategies but having similar sequencing depth values (20 sequencing datasets for 8 different species; Tables S1 and S2) and (ii) from the same sequencing platform and strategy but exhibiting different sequencing depth (30 sequencing datasets for 12 different species; Tables S1 and S2).

BR and OBRA annotations were compared to REF annotations based on the number and length of predicted protein sequences. We also compared sequence similarity between the predicted BR or OBRA proteins and the REF proteins using cd‐hit v4.8.1 [39], which allowed us to group into ‘clusters’ all the sequences sharing at least 90% sequence similarity over a minimum of 50% of the shortest sequence. In addition the completeness of BR, OBRA and REF annotations were compared using Busco v5.7.1.

### 2.8 Analysing Proteomics Data Using REF, BR and OBRA Annotations

Mass spectrometry data were downloaded from datasets available in the PRIDE repository [40] for 27 species (Tables S4 and S5). Only datasets acquired on high resolution instruments were selected (Q‐Orbitrap, tribrid and Q‐TOF instruments). The acquisition method (essentially DDA) and eventual quality controls that were used to generate these datasets are given in Table S4. They were processed using MaxQuant v2.0.3.1 [41]. Peaklists were searched using Andromeda search engine implemented in MaxQuant. The protein database contained merged protein sequences from REF, BR and OBRA annotations (with or without discarding small proteins of less than 100 amino acids), which allowed us to determine whether protein identification was made from REF and/or BR and/or OBRA entries. Sequences of common contaminants (247 entries; contaminants.fasta included in MaxQuant), as well as decoy sequences (revert mode) were then added. The first search was performed using a precursor mass tolerance of 20 ppm. Fragment ion mass tolerance was set to 20 ppm. The second peptide research option was enabled. Carbamidomethylation of cysteines was considered as a fixed modification and oxidation of methionines and acetylation of protein N‐termini as variable modifications during the search. A maximum number of two missed cleavages was tolerated, and a false discovery rate (FDR) of 1% for both peptide spectrum matches (minimum length of seven amino acids) and proteins was accepted during identification. All other parameters were set as default. In addition we also compared the length of identified proteins depending on the annotation of origin.

## 3 Results and Discussion

### 3.1 Brownotate Generates Fragmented, Slightly Less Complete but Good Quality Assemblies

As shown in Table 1, DNA assemblies generated using Brownotate are generally similar in length to REF assemblies, except for six of the nine plants where they are 1.6–4 times longer. The N50 was generally much lower for BR assemblies (especially for eukaryotes) than for REF assemblies, indicating greater fragmentation (Table 1). The difference is potentially due to the precise adjustment of the assembly parameters and the data curation carried out by NCBI experts for the REF assemblies. It may also be that different sequencing datasets may have been used to generate the REF and BR assemblies, with potentially less informative datasets used here with Brownotate compared to those used by NCBI experts for REF assemblies. The sequencing platform or strategy directly influences the characteristics of a sequencing dataset (e.g., read length, genome coverage), thus probably the capacity of assemblers to perform correctly. We observed that when comparing the BR assemblies obtained from sequencing datasets of similar depth for a given species, the use of short‐read WGS data generally provides assemblies with greater N50 values, Busco completeness and coverage of REF assemblies by BR assemblies in comparison with the use of long‐read WGS data or short‐read non‐WGS data (Figure S1 and Table S6). It may be surprising that using sequencing datasets from long‐read platforms does not yield the best results, but this can be explained by the use of Megahit in Brownotate, which is known to not perform well with this type of data [30]. This is why Brownotate is parameterized to favour the selection of short‐read WGS data.

**TABLE 1:** Characteristics of reference (REF) and Brownotate‐derived (BR) genome assemblies.

Species		Assembly length (bp)	N50 (bp)	Coverage (% on REF)	No. of BUSCO groups	
					*N* (%)	Overlap
*S. aureus* **(Ba_1_)**	**REF**	2,821,361	2,821,361	94.1	447 (99)	445
	**BR**	2,862,830	1,446,283		448 (100)	
*L. brevis* **(Ba_2_)**	**REF**	2,552,671	2,552,671	83.5	400 (100)	320
	**BR**	3,760,101	2689		321 (80)	
*M. xanthus* **(Ba_3_)**	**REF**	9,139,763	9,139,763	99.6	122 (98)	122
	**BR**	9,538,195	801,254		122 (98)	
*E. nidulans* **(Fu_1_)**	**REF**	30,275,969	2,478,513	99.1	3969 (95)	3945
	**BR**	41,795,055	65,854		3971 (95)	
*S. cerevisiae* **(Fu_2_)**	**REF**	12,157,105	924,431	96.6	2129 (100)	2126
	**BR**	14,120,026	75,262		2126 (100)	
*F. oxysporum* **(Fu_3_)**	**REF**	47,906,303	4,457,292	87.5	4393 (98)	4381
	**BR**	82,120,551	102,910		4410 (98)	
*A. bisporus* **(Fu_4_)**	**REF**	30,233,745	2,334,609	87.7	3595 (93)	3557
	**BR**	29,932,517	149,140		3620 (93)	
*B. botryosum* **(Fu_5_)**	**REF**	46,674,321	444,941	95.8	2540 (88)	2427
	**BR**	44,793,876	19,490		2449 (84)	
*R. toruloides* **(Fu_6_)**	**REF**	20,223,942	574,942	99.9	1552 (88)	1543
	**BR**	20,376,809	165,092		1548 (88)	
*D. melanogaster* **(Ar_1_)**	**REF**	143,726,002	25,286,936	86.3	3243 (99)	3175
	**BR**	150,572,795	11,320		3182 (97)	
*A. gambiae* **(Ar_2_)**	**REF**	265,027,044	49,364,325	88.7	3174 (97)	2791
	**BR**	413,202,482	1907		2854 (87)	
*A. mellifera* **(Ar_3_)**	**REF**	225,250,884	13,619,445	97.4	5854 (98)	5477
	**BR**	284,756,162	13,780		5509 (92)	
*G. gallus* **(Bi_1_)**	**REF**	1,053,332,251	90,861,225	94.8	8062 (97)	7120
	**BR**	1,015,205,309	43,167		7183 (86)	
*C. caeruleus* **(Bi_2_)**	**REF**	1,186,980,630	16,846,143	91.7	10,341 (95)	8674
	**BR**	1,115,283,351	40,057		8804 (81)	
*S. demersus* **(Bi_3_)**	**REF**	1,275,043,525	15,386,364	97.7	8002 (96)	6548
	**BR**	1,298,614,991	28,410		6672 (80)	
*N. naja* **(Re_1_)**	**REF**	1,768,535,092	224,088,900	89.9	6741 (90)	2601
	**BR**	2,195,289,329	4253		2705 (36)	
*P. vitticeps* **(Re_2_)**	**REF**	1,716,675,060	2,477,614	93.3	7054 (94)	2242
	**BR**	2,058,457,618	3924		2278 (30)	
*C. caretta* **(Re_3_)**	**REF**	2,134,012,717	130,956,235	94.5	7188 (96)	3361
	**BR**	2,384,262,771	9709		3439 (46)	
*C. sabateus* **(Ma_1_)**	**REF**	2,937,827,970	81,790,585	93.6	13,093 (95)	6694
	**BR**	3,215,377,518	21,130		6763 (49)	
*P. cinereus* **(Ma_2_)**	**REF**	3,192,581,492	11,587,828	98.2	8673 (94)	6119
	**BR**	3,375,561,314	45,993		6227 (68)	
*U. arctos* **(Ma_3_)**	**REF**	2,474,258,672	70,076,652	93.1	13,849 (96)	8999
	**BR**	2,468,164,009	34,321		9057 (62)	
*C. lupus* **(Ma_4_)**	**REF**	2,481,983,352	64,299,765	93.6	3273 (98)	1874
	**BR**	2,404,979,967	29,401		1992 (56)	
*O. orca* **(Ma_5_)**	**REF**	2,647,351,467	114,219,206	76.0	3251 (97)	455
	**BR**	2,116,029,905	2184		489 (14)	
*S. suricatta* **(Ma_6_)**	**REF**	2,353,578,744	141,453,419	98.4	3222 (96)	1886
	**BR**	2,654,146,290	24,022		2006 (58)	
*C. asiatica* **(Ma_7_)**	**REF**	4,210,110,458	13,470,186	63.8	3253 (97)	842
	**BR**	2,243,934,135	1702		931 (25)	
*B. taurus* **(Ma_8_)**	**REF**	2,770,686,120	103,308,737	73.0	3261 (97)	649
	**BR**	2,411,338,559	1670		682 (20)	
*D. leucas* **(Ma_9_)**	**REF**	2,362,774,659	31,183,418	98.6	3241 (97)	2135
	**BR**	2,442,816,188	34,734		2254 (64)	
*O. aries* **(Ma_10_)**	**REF**	2,654,063,983	101,274,418	95.6	3257 (97)	1623
	**BR**	2,744,872,742	9884		1703 (49)	
*P. tigris* **(Ma_11_)**	**REF**	2,408,695,688	146,942,463	98.2	3272 (98)	1751
	**BR**	2,430,665,556	29,217		1807 (50)	
*P. promelas* **(Fi_1_)**	**REF**	1,066,429,022	11,952,773	68.5	3526 (97)	1128
	**BR**	750,862,167	2692		1136 (31)	
*D. rerio* **(Fi_2_)**	**REF**	1,679,203,469	52,186,027	72.1	3496 (96)	1375
	**BR**	1,520,659,234	7555		1391 (38)	
*O. latipes* **(Fi_3_)**	**REF**	734,057,086	31,218,526	92.5	3525 (97)	2537
	**BR**	900,523,275	9262		2560 (70)	
*C. sativa* **(Pl_1_)**	**REF**	876,147,649	91,913,879	62.8	1535 (95)	1288
	**BR**	1,389,773,526	358		1340 (83)	
*A. thaliana* **(Pl_2_)**	**REF**	119,668,634	23,459,830	97.3	4560 (99)	4538
	**BR**	205,390,114	42,256		4539 (99)	
*R. chinensis* **(Pl_3_)**	**REF**	515,118,979	69,643,165	87.4	1597 (99)	1094
	**BR**	1,910,598,207	427		1101 (68)	
*B. distachyon* **(Pl_4_)**	**REF**	271,298,618	59,130,575	98.7	4805 (98)	4388
	**BR**	851,823,047	490		4519 (90)	
*H. vulgare* **(Pl_5_)**	**REF**	4,225,713,981	610,333,535	81.5	4788 (98)	4194
	**BR**	5,036,979,044	1042		4355 (86)	
*V. vinifera* **(Pl_6_)**	**REF**	495,807,417	26,899,771	91.1	1587 (98)	1003
	**BR**	1,801,822,283	489		1067 (63)	
*C. annuum* **(Pl_7_)**	**REF**	3,212,488,018	227,195,441	81.4	1558 (97)	1149
	**BR**	3,811,482,623	8923		1196 (72)	
*P. aphrodite* **(Pl_8_)**	**REF**	1,025,096,742	946,429	81.6	2960 (92)	679
	**BR**	4,130,467,851	347		707 (21)	
*L. ruthenicum* **(Pl_9_)**	**REF**	2,251,296,259	188,110,659	78.4	1597 (99)	496
	**BR**	2,571,561,736	942		560 (31)	
*P. falciparum* **(Ot_1_)**	**REF**	23,326,872	1,687,656	86.7	3594 (99)	3202
	**BR**	23,797,790	5141		3212 (88)	
*A. queenslandica* **(Ot_2_)**	**REF**	157,519,205	123,180	58.8	845 (89)	498
	**BR**	217,509,282	850		523 (55)	
*C. elegans* **(Ot_3_)**	**REF**	100,286,401	17,493,829	94.2	3092 (99)	2911
	**BR**	122,229,425	37,775		2913 (93)	

*Note*: Various metrics were used to compare REF to BR DNA assemblies, including their length (cumulative length of all contigs), quality evaluated using the N50 (shortest contig length to be included to cover the first half of an assembly after all contigs have been sorted by decreasing size) and the proportion of the REF assemblies covered by the BR assemblies. The completeness of the assemblies was estimated using sets of Benchmarking Universal Single‐Copy Orthologs (BUSCO) with the number (*N*) of BUSCO orthologs indicated along with the fraction of the theoretical number it represents (in % in brackets) and the number of overlapping BUSCO orthologs between REF and BR assemblies. For each species, an abbreviation is defined between bracket, which is reused as an identifier in Figures 2 and 3.

Abbreviations: Ar, arthropods; Ba, bacteria; Bi, birds; Fi, fish; Fu, fungi; Ma, mammals; Ot, other taxonomic classes; Pl, plants; Re, reptiles.

Another reason for fragmentation may be that we used datasets with low sequencing depths, resulting in gaps in the assembly [42]. Table S7 shows that when comparing BR assemblies obtained from the same sequencing platform or strategy for a given species, the sequencing depth does not influence the coverage of REF assemblies by BR assemblies. However, the sequencing depth required to obtain maximal N50 values appears to be between 50× and 150×. For example, the N50 decreases for *A. thaliana* (−24%) when using a dataset with a depth of 507x compared to a dataset with a depth of 68x, or for *P. cinereus* (−94%) when using a dataset with a depth of 7× depth compared to a dataset with a depth of 75× (Table S7). This is in line with a previous work showing that the 50× depth range is optimal for various assemblers [43]. This is why Brownotate is parameterized to select sequencing datasets in the 50×–100× range.

The longer assemblies and extreme fragmentation observed for plants could be due to the large number of repetitive DNA sequences in their genomes [43], which makes assembly very difficult thus causing misassembled rearrangements [44]. The six plant species for which BR assemblies were particularly longer than REF assemblies are also those exhibiting the lowest N50 values (Table 1). This clearly highlights a close link between fragmentation and the length of assemblies. Like plants, but to a lesser extent, mammals, reptiles and fish also have numerous sequence repeats in their genomes, while birds have far fewer [43, 45]. Their N50 is indeed lower for BR assemblies than for REF assemblies, but to a lesser extent if we refer to the results obtained for plants (Table 1). However, Busco scores are higher in plants than in reptiles, mammals and fish (68% vs. 45% on average, Table 1). This suggests that even with highly fragmented assemblies, plant genes are better predicted than those of mammals, reptiles and fish. The shorter introns in plants compared with animals [43] might have facilitated gene prediction for plants despite fragmentation. It is known that the use of short‐read data makes the assembly of genomes difficult [44]. We believe that implementing Brownotate with an assembler capable of handling long‐read data will improve the assembly of repeat‐rich genomes in the future.

Interestingly, the coverage of REF assemblies by BR assemblies was quite good despite highly fragmented assemblies obtained with Brownotate, even for plants. Indeed, coverage was greater than 95% for 14 species of the 44 species, 90% for 11 other species, 80% for 10 other species and 70% for 5 other species. For the four remaining species, values between 58% and 69% were obtained.

As expected, Busco completeness (as a percentage of expected Busco orthologs) was high for REF assemblies, with values above 95% for 35 of 44 assemblies and above 88% for the 9 other ones (Table 1). On the other hand, BR assemblies appear to be slightly less complete, with only 11 out of 44 assemblies having values above 90%, 10 other ones having values above 80%. For the remaining 23 BR assemblies, notably those for reptiles, mammals and fish, Busco completeness is between 14% and 72%. These three last taxonomic classes are among those where the expected number of Busco orthologs [46] is highest, which may argue in favour of a greater difficulty in expecting completeness during assembly. As for fragmentation (see above), the use of insufficiently informative sequencing datasets could perhaps be blamed here. We can nevertheless note the high completeness for the three bird species, which are also among the taxonomic classes with numerous expected Busco orthologs. This could reflect the fact that we used only short‐read WGS data (see above) for birds or that Brownotate performs particularly well to assemble their genome. Next, we postulated that the issue of fragmentation (notably the fragmentation of orthologs) would be more pronounced for long genomes, and we could confirm that the longer the assembly, the more challenges Brownotate faces in terms of Busco completeness. Indeed, a significant negative correlation was found between the percentage of expected Busco orthologs from BR assemblies and the length of REF assemblies (Figure S2).

### 3.2 Brownotate Generates Good Quality Annotations, Albeit With an Excess of Predicted Sequences

**FIGURE 2:**
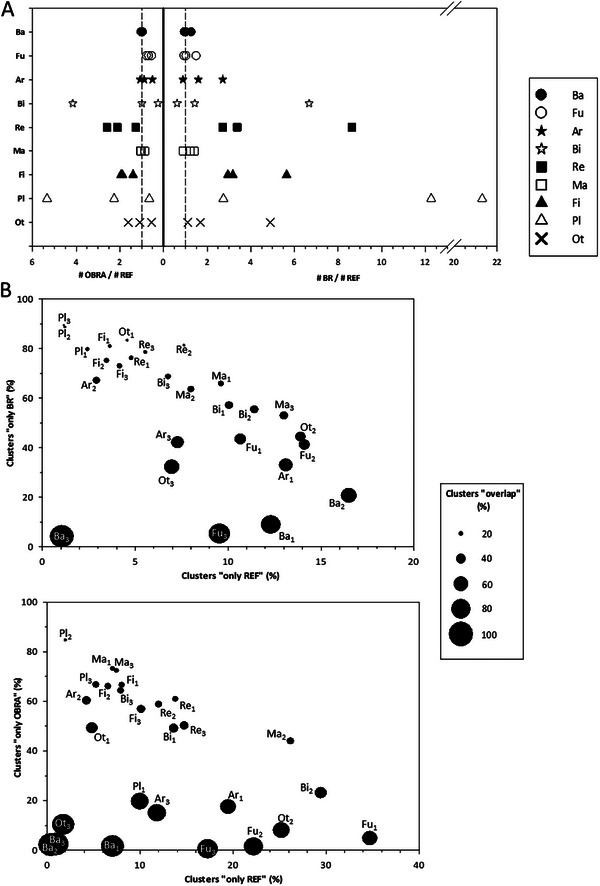
Predicted protein sequences in the reference (REF) and Brownotate (BR and OBRA) annotations. (A) Ratio of the number of protein sequences predicted by Brownotate (OBRA or BR) over the number in the reference (REF) annotations. Numbers of predicted proteins are detailed in Table S5. (B) Proportion of protein clusters containing only REF, BR or OBRA predicted sequences, or containing both BR and REF or both OBRA and REF sequences (“overlap”). Species are defined using abbreviations as reported in Table 1. Ar indicates arthropods; Ba, bacteria; Bi, birds; Fi, fish; Fu, fungi; Ma, mammals; Ot, other taxonomic classes; Pl, plants; Re, reptiles.

As shown in Figure 2A, the number of protein‐coding sequences predicted in the BR annotations was roughly similar to that in the REF annotations in most cases for bacteria, fungi and mammal species, but was generally greater for the others, especially reptiles, fish and plants (7 ± 2 times, up to 21 times for *C. sativa*). Improved results were obtained in OBRA annotations, the number of predicted protein sequences reaching values most often closer to that in REF annotations (Figure 2A). It is, therefore, the assembly step in Brownotate that seems responsible for the differences we observe with REF datasets, which could be the result of fragmentation (see above), and also of longer assemblies with Brownotate, particularly in the case of plants (see above). Few parameters in the annotation step may also have played a role here, such as the ‘minmeanexonintronprob’ parameter in Augustus, that is, set to a low default value (0.4), which is a permissive probability value for considering a region as an exon or intron. The same applies to the ‘genemodel’ parameter set to ‘partial’, enabling the prediction of partial genes. The question of whether these excess proteins are real or not will be examined below.

To further compare the different annotations, we then assessed their redundancy using cd‐hit v4.8.1 (Figure 2B and Table S8). A first important observation is the low proportion of clusters that contain only REF proteins (1%–17% when comparing REF to BR annotations, 0%–35% when comparing REF to OBRA annotations), suggesting good overall predictive quality for BR and OBRA annotations with only a few missed proteins whatever the taxonomic class considered. A very good overlap between REF and BR or OBRA annotations was found for bacteria and to a lesser extent for fungi, that is, those with the shorter assemblies (Table 1). Consistent with the greater number of protein‐coding sequences predicted with Brownotate (see above), a larger proportion of clusters containing only BR proteins (64% ± 4% for all species except bacteria and *Saccharomyces cerevisiae*) or only OBRA proteins (57% ± 5% for plants, reptiles, fish, mammals and birds) was found. Whether these excess protein sequences were missed in REF annotations or wrongly predicted by Brownotate will be examined below.

The size distribution of predicted proteins broadly followed a comparable profile in REF, BR and OBRA annotations, with, on average, most predicted proteins (91% ± 1%) being less than 900 amino acids long, proportions then gradually decreasing with protein length to reach the lower values (0.68% ± 0.04%) in the 1000–2000 amino acid range and increasing slightly (up to 1.3% ± 0.3%) above the 2000 amino acid length (Figure S3). Closer examination revealed a higher proportion of shorter proteins in OBRA annotations, with 90% of predicted sequences being up to only 600–900 amino acids long (up to 1100 amino acids for arthropods). This trend was even more pronounced in BR annotations, with 90% of predicted sequences being up to only 300–700 amino acids long. The Brownotate's tendency to predict shorter sequences could be the result of fragmented assemblies and/or non‐stringent parameterisation of Augustus (see above). The strong overlap between the three types of annotation in terms of sequence clusters (see above) supports the hypothesis that these short sequences correspond to partial sequences. Alternatively, such short sequences could correspond to so‐called small proteins [47], which sequences are not often found in classic databases such as UniprotKB [48].

Concerning plants, 1000 amino acids was the upper limit for the size of 90% of the predicted proteins in the REF annotation, and the limit dropped to 700 amino acids in the OBRA annotation and 300 amino acids in the BR annotation. Brownotate also tended to predict a lower proportion of proteins over 2000 amino acids compared to OBRA (5 ± 1 times lower) and especially REF (24 ± 10 times lower) annotations.

As shown in Table 2, the Busco completeness (as a percentage of expected Busco orthologs) was high for REF annotations (97% ± 1%) and slightly lower for OBRA annotations (82% ± 2%) and BR annotations (68% ± 5%). The lower completeness of the BR annotations appears mainly due to the values for reptiles (30% ± 5%), fish (43% ± 12%) and mammals (46% ± 5%). Completeness, therefore, follows the same overall trend for annotations as for assemblies (see above). If we look in more detail, completeness of REF annotations was in most cases similar to that of REF assemblies (ratio annotation/assembly = 1.00 ± 0.01), while it was lower in BR annotations than in BR assemblies (ratio annotation/assembly = 0.90 ± 0.02). Although the values are very good in most cases, this may reflect that the annotation step in Brownotate could be further optimized, for example, through a more species‐specific parametrisation of Augustus by providing more than only one extrinsic dataset to better train the HMM model [14]. Interestingly, it can be seen that Brownotate provides a complementarity, that is, admittedly low but most often not zero compared with the REF annotations, with OBRA and BR annotations specifically bringing 1.4% ± 0.5% of Busco orthologs (up to 15% for *Spheniscus demersus* and *Naja naja*; Table 2).

**TABLE 2:** Characteristics of reference (REF), Brownotate‐derived (BR and BRAO) annotation datasets.

Species	BUSCO lineage (no. of groups)	No. of BUSCO groups (% of total)			No. of BUSCO group overlap			
		REF	OBRA	BR	REF and OBRA	REF and BR	OBRA and BR	All three datasets
*S. aureus* **(Ba_1_)**	bacillales_odb10 (450)	445 (99%)	447 (99%)	448 (100%)	445	443	445	443
*L. brevis* **(Ba_2_)**	lactobacillales_odb10 (402)	400 (100%)	400 (100%)	279 (69%)	400	278	278	278
*M. xanthus* **(Ba_3_)**	bacteria_odb10 (124)	122 (98%)	122 (98%)	122 (98%)	122	122	122	122
*E. nidulans* **(Fu_1_)**	eurotiales_odb10 (4191)	3797 (91%)	2400 (57%)	3471 (83%)	2275	3244	2323	2213
*S. cerevisiae* **(Fu_2_)**	saccharomycetes_odb10 (2137)	2129 (100%)	1927 (90%)	2088 (98%)	1926	2088	1919	1919
*F. oxysporum* **(Fu_3_)**	hypocreales_odb10 (4494)	4478 (100%)	4259 (95%)	4357 (97%)	4247	4344	4220	4208
*D. melanogaster* **(Ar_1_)**	diptera_odb10 (3285)	3285 (100%)	3124 (95%)	3110 (95%)	3124	3110	3067	3067
*A. gambiae* **(Ar_2_)**	diptera_odb10 (3 285)	3235 (97%)	2923 (89%)	2668 (81%)	2863	2597	2538	2489
*A. mellifera* **(Ar_3_)**	hymenoptera_odb10 (5 991)	5923 (99%)	5493 (92%)	5320 (89%)	5452	5270	5093	5056
*G. gallus* **(Bi_1_)**	vertebrata_odb10 (8 338)	8285 (99%)	6238 (75%)	6486 (78%)	6222	6463	5262	5247
*C. caeruleus* **(Bi_2_)**	passeriformes_odb10 (10 844)	10,624 (98%)	7708 (71%)	7100 (65%)	7640	6987	6145	6096
*S. demersus* **(Bi_3_)**	vertebrata_odb10 (8 338)	6595 (79%)	6325 (76%)	4983 (60%)	5177	4092	4622	3839
*N. naja* **(Re_1_)**	sauropsida_odb10 (7 480)	5295 (71%)	4876 (65%)	2106 (28%)	3914	1800	1661	1496
*P. vitticeps* **(Re_2_)**	sauropsida_odb10 (7 480)	7285 (97%)	5020 (67%)	1612 (22%)	4995	1603	1480	1476
*C. caretta* **(Re_3_)**	sauropsida_odb10 (7 480)	7371 (99%)	5062 (68%)	2903 (39%)	5050	2862	2644	2636
*C. sabateus* **(Ma_1_)**	primates_odb10 (13 780)	13,608 (99%)	8892 (64%)	5243 (38%)	8864	5218	4479	4467
*P. cinereus* **(Ma_2_)**	mammalia_odb10 (9 226)	9053 (98%)	6328 (69%)	5070 (55%)	6308	5051	4296	4285
*U. arctos* **(Ma_3_)**	carnivora_odb10 (14 502)	14,392 (99%)	9810 (68%)	6696 (46%)	9762	6665	6065	6040
*P. promelas* **(Fi_1_)**	actinopterygii_odb10 (3 640)	3595 (99%)	2850 (78%)	1077 (30%)	2845	1072	950	949
*D. rerio* **(Fi_2_)**	actinopterygii_odb10 (3 640)	3596 (99%)	2758 (76%)	1173 (32%)	2745	1168	1086	1085
*O. latipes* **(Fi_3_)**	actinopterygii_odb10 (3 640)	3587 (99%)	2823 (78%)	2393 (66%)	2816	2371	2091	2086
*C. sativa* **(Pl_1_)**	embryophyta_odb10 (1 614)	1541 (96%)	1357 (84%)	1273 (79%)	1353	1227	1123	1122
*A. thaliana* **(Pl_2_)**	brassicales_odb10 (4 596)	4596 (100%)	4460 (97%)	4478 (97%)	4460	4478	4421	4421
*R. chinensis* **(Pl_3_)**	embryophyta_odb10 (1 614)	1608 (100%)	1348 (84%)	936 (58%)	1348	936	858	858
*P. falciparum* **(Ot_1_)**	plasmodium_odb10 (3 642)	3610 (99%)	3566 (98%)	3187 (88%)	3562	3183	3160	3157
*A. queenslandica* **(Ot_2_)**	metazoa_odb10 (954)	866 (91%)	811 (85%)	501 (52%)	799	478	456	451
*C. elegans* **(Ot_3_)**	nematoda_odb10 (3 131)	3131 (100%)	2915 (93%)	2843 (91%)	2915	2843	2736	2736

*Note*: We used species‐specific Busco lineages to determine the number of expected of universal single‐copy orthologs and enable comparison of REF to BR and OBRA annotations, the number of theoretical Busco orthologs being indicated in brackets. The completeness of the annotations was estimated with the number of BUSCO orthologs indicated along with the fraction of the theoretical number it represents (in % in brackets) and the number of overlapping BUSCO orthologs between REF, OBRA and BR annotations. Species are defined using abbreviations (between brackets) as reported in Table 1.

Abbreviations: Ar, arthropods; Ba, bacteria; Bi, birds; Fi, fish; Fu, fungi; Ma, mammals; Ot, other taxonomic classes; Re, reptiles; Pl, plants.

### 3.3 Brownotate Generates Excellent Protein Sequence Databases for Proteomics Data Analysis

**FIGURE 3:**
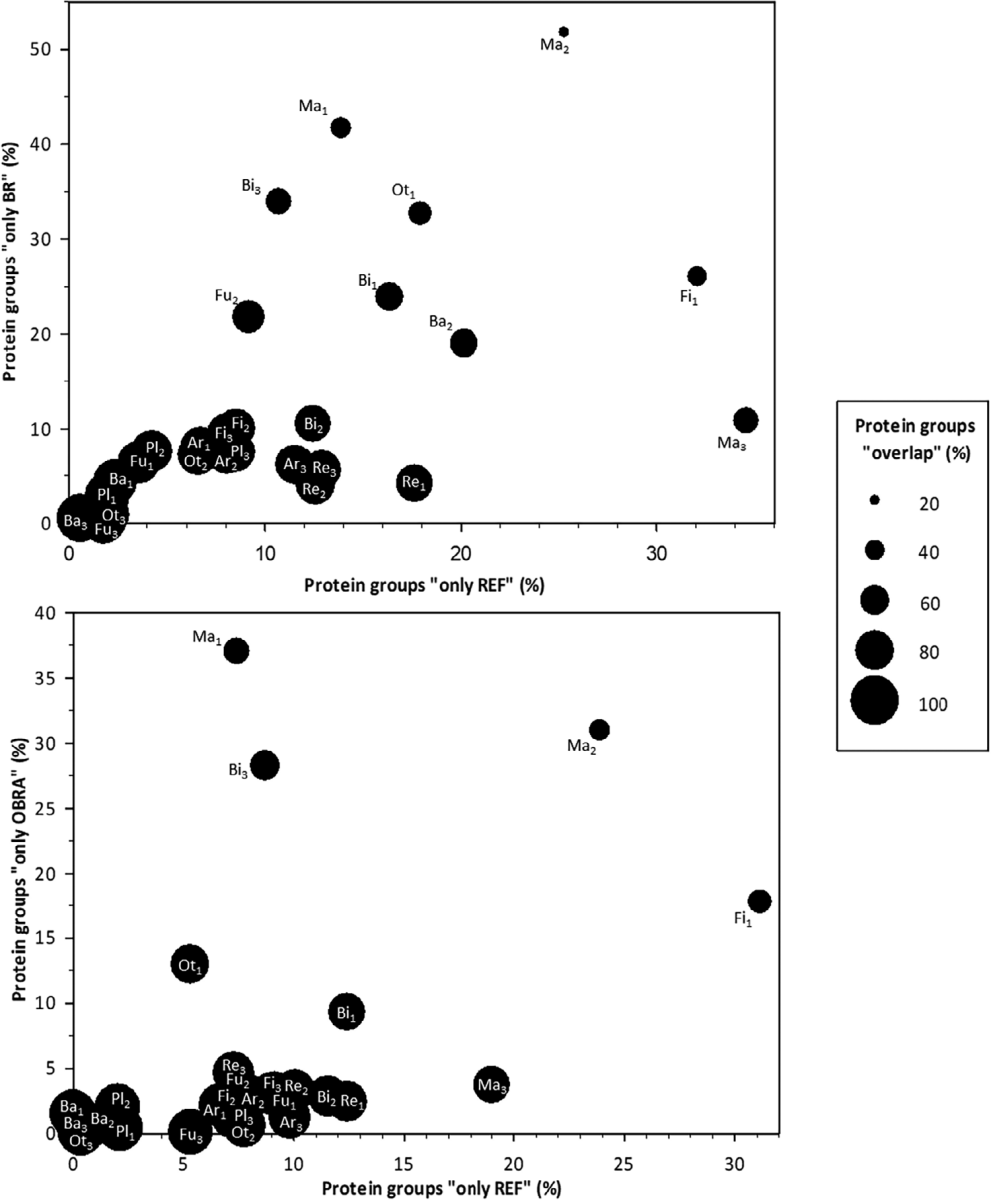
Protein groups identified by MaxQuant when using the reference (REF) and Brownotate (BR and OBRA) predicted protein sequences. The proportion of protein clusters containing protein groups identified only when using REF, BR or OBRA predicted protein sequences, or containing both BR and REF or OBRA and REF protein sequences (“overlap”) were computed from the actual numbers of identified protein groups as detailed in Table S6. Species are defined using abbreviations as reported in Table 1. Ar indicates arthropods; Ba, bacteria; Bi, birds; Fi, fish; Fu, fungi; Ma, mammals; Ot, other taxonomic classes; Pl, plants; Re, reptiles.

Using the REF protein databases and those generated using Brownotate to analyse real proteomic data for 27 species revealed a very similar number of protein groups identified regardless of the database, as well as a high overlap up to 86% ± 14% between REF and OBRA and 77% ± 19% between REF and BR (Figure 3 and Table S9). The size distribution of the identified proteins (Figure S4) generally followed a comparable profile regardless of the protein database used (REF, BR or OBRA). However, a greater proportion of small proteins, notably below 100 amino acids, were identified when using BR and, to a lesser extent, OBRA annotations. It may be that the prediction of partial proteins explains these results. As detailed in Table S9, filtering out proteins of less than 100 amino acids from sequence databases did not drastically change the total number of identified proteins, but a slight downward trend (3.5% on average) was observed for half of the species, an upward trend being observed for the other half (especially marked for *Caretta caretta* and *C. sativa*, 200% on average, Figure S5A,B). This would corroborate the hypothesis that the proteins in excess in the Brownotate annotations (see above) are, for the most part, incorrectly or erroneously predicted. This result would justify discarding short sequences in BR and OBRA annotations.

Using the REF databases specifically allowed identification of protein groups not identified using the OBRA (8% ± 7%) or BR (11% ± 9%) databases. That said, the reverse is true since protein groups are also specifically identified when using the OBRA (6% ± 10%) or BR (12% ± 13%) database compared with REF. It should be noted that when the overlap is less good (< 60%), for example, if we compare REF and BR for reptiles, for *S. demersus*, *D. rerio* and *Amphimedon queenslandica*, the percentages of protein groups specifically identified by REF and also by BR are both increased. This is also true for *N. naja* and *C. caretta* and *D. rerio* when comparing REF and OBRA. So, not only do the databases generated by Brownotate (OBRA and BR) make it possible to identify the vast majority of the protein groups identified with the REF database while ‘missing’ a minimum of them, but they also provide information that the REF database does not provide, for example, about possible still not predicted small proteins (see above). Filtering out proteins of less than 100 amino acids from sequence databases induced a slight increase in the proportions of proteins identified solely with the REF protein database (up to 10% for *D. rerio* and *A. queenslandica*) and a slight decrease in the proportions of proteins identified solely with the BR protein database (down to −10% for *A. queenslandica*) or in an overlapping manner (−11% for *C. sativa*) (Figure S5C,D). The difference was less pronounced when filtering the OBRA database. It is also interesting to note that filtering generally tended to allow identifying proteins with, on average, 4%–10% more peptides per protein, whatever the database from which proteins were identified (BR/OBRA, REF or both; Figure S5E,F), which is expected to increase the confidence in identifications. The oversized databases due to the excess of proteins predicted by OBRA and especially BR, therefore, certainly prevent the identification of certain REF proteins and limit sequence coverage of identified proteins. Although Brownotate annotations allow an overall satisfying analysis of proteomics data, removing small predicted proteins should be considered, an option, that is, offered in Brownotate's user interface.

## 4 Concluding Remarks

The overall quality of the assemblies and annotations produced using Brownotate appears to be good for most of the taxonomic class considered. It is important to note that some of Brownotate's limitations, such as assembly fragmentation or the prediction of excess protein sequences, may slightly impair the quality of protein identifications compared to the use of REF protein databases when interpreting proteomics data. Removing small proteins from sequence databases generated by Brownotate may help resolve this issue. Moreover, the future implementation of other assemblers able to deal with long reads, such as Flye or Raven, should help improve the assembly of repeat‐rich genomes. More sophisticated parametrisation of Brownotate (e.g., notably for Augustus) will also be allowed in the future to refine the results.

By making it possible to easily generate a good quality protein sequence database for any species, either after automatic exploration of existing protein sequences, or after assembly and/or annotation of available DNA sequences, Brownotate will expand the possibilities for analysing the proteome of a growing number of model organisms and for optimizing the analysis to the particular proteome of given individuals. Benefits are, therefore, strongly expected in the fields of ecology/ecophysiology and evolutionary biology, as well as for personalised medicine. In addition, one of the first improvements we will make to Brownotate in the near future will be the implementation of the ability to perform an annotation from RNAseq data.

## Conflicts of Interest

The authors declare no conflicts of interest.

## Supporting information




**Supporting File 1**: pmic70094‐sup‐0001‐FiguresS1‐S5.pdf.

**Supporting File 2**: pmic70094‐sup‐0002‐Tables.pdf.

**Supporting File 3**: pmic70094‐sup‐0003‐TableS1.xlsx.

**Supporting File 4**: pmic70094‐sup‐0004‐TableS5.xlsx.

**Supporting File 5**: pmic70094‐sup‐0005‐TableS6.xlsx.

**Supporting File 6**: pmic70094‐sup‐0006‐TableS7.xlsx.

**Supporting File 7**: pmic70094‐sup‐0007‐TableS9.xlsx.

## Data Availability

The Brownotate pipeline and the web interface are open‐source and available at https://github.com/LSMBO/Brownotate and https://github.com/LSMBO/brownotate‐app.
