# Supplementary material for: Brownotate, a Comprehensive Solution to Generate Protein Sequence Databases for Any Species
Source: Proteomics. 2026 Jan 6;26(5):13–26. doi: 10.1002/pmic.70094 (PMC13106930; doi:10.1002/pmic.70094)
Supplement: Supplementary file 1 — Supporting File 1: pmic70094‐sup‐0001‐FiguresS1‐S5.pdf. [file PMIC-26--s003.pdf]

Brownotate, a comprehensive solution to generate protein sequence databases for any species

Adrien Brown[1,2], Alexandre Burel[1,2], Sarah Cianférani[1,2], Christine Carapito[1,2], Fabrice Bertile[1,2]

[1] Université de Strasbourg, CNRS, IPHC UMR7178, Laboratoire de Spectrométrie de Masse BioOrganique (LSMBO), 25 rue Becquerel, 67087 Strasbourg, France.

[2] Infrastructure Nationale de Protéomique ProFI – FR2048, 67087 Strasbourg, France.

Supplementary figures

Figure S1. Influence of the type of sequencing datasets on the quality of Brownotate assembly

Figure S2. Completeness of DNA assemblies according to their length

Figure S3. Size distribution of predicted proteins in the reference (REF) and Brownotate (BR and OBRA) annotations

Figure S4. Size distribution of the proteins identified by MaxQuant when using the reference and Brownotate annotations

Figure S5. MaxQuant results obtained with or without discarding small proteins of less than 100 amino acids in protein sequence databases

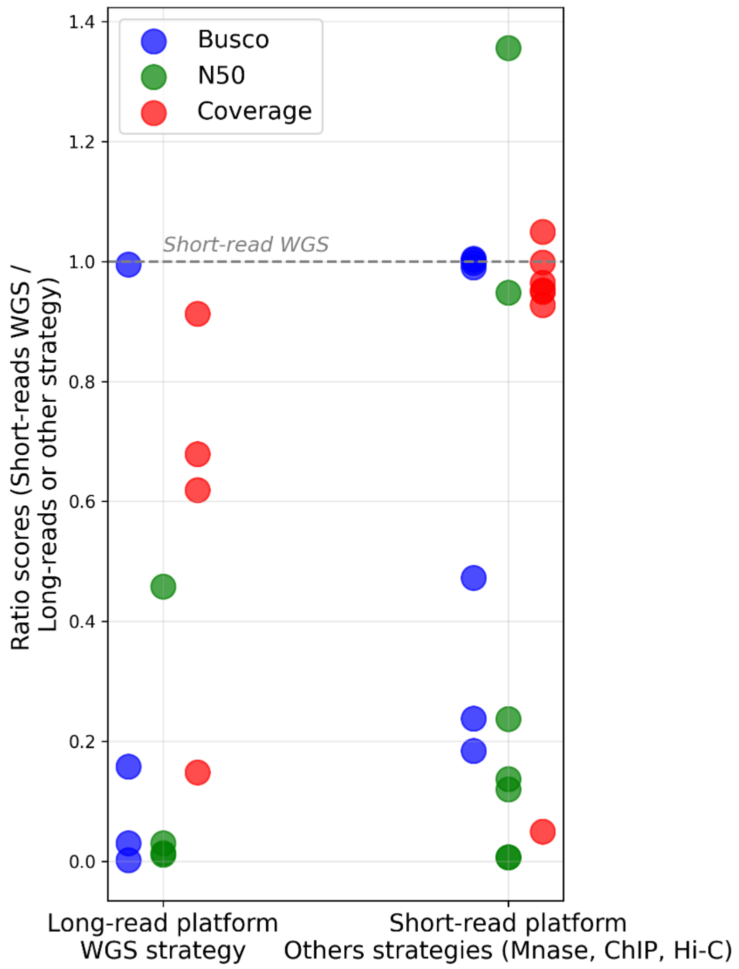

Figure S1. Influence of the type of sequencing datasets on the quality of Brownotate assembly

Brownotate assemblies were generated using short-read WGS data for eight species. For the same species, other assemblies were produced with either short-read data other than WGS (e.g. MNase, ChIP, Hi-C), or long-read data (See Table S1). For each assembly obtained from sequencing data other than short-read WGS data, we calculated the ratio between the Busco score (blue), N50 (green) or coverage of REF assemblies by BR assemblies (red) and the corresponding values obtained from short-read WGS data. Exact values and dataset details are provided in Supplementary Table S6.

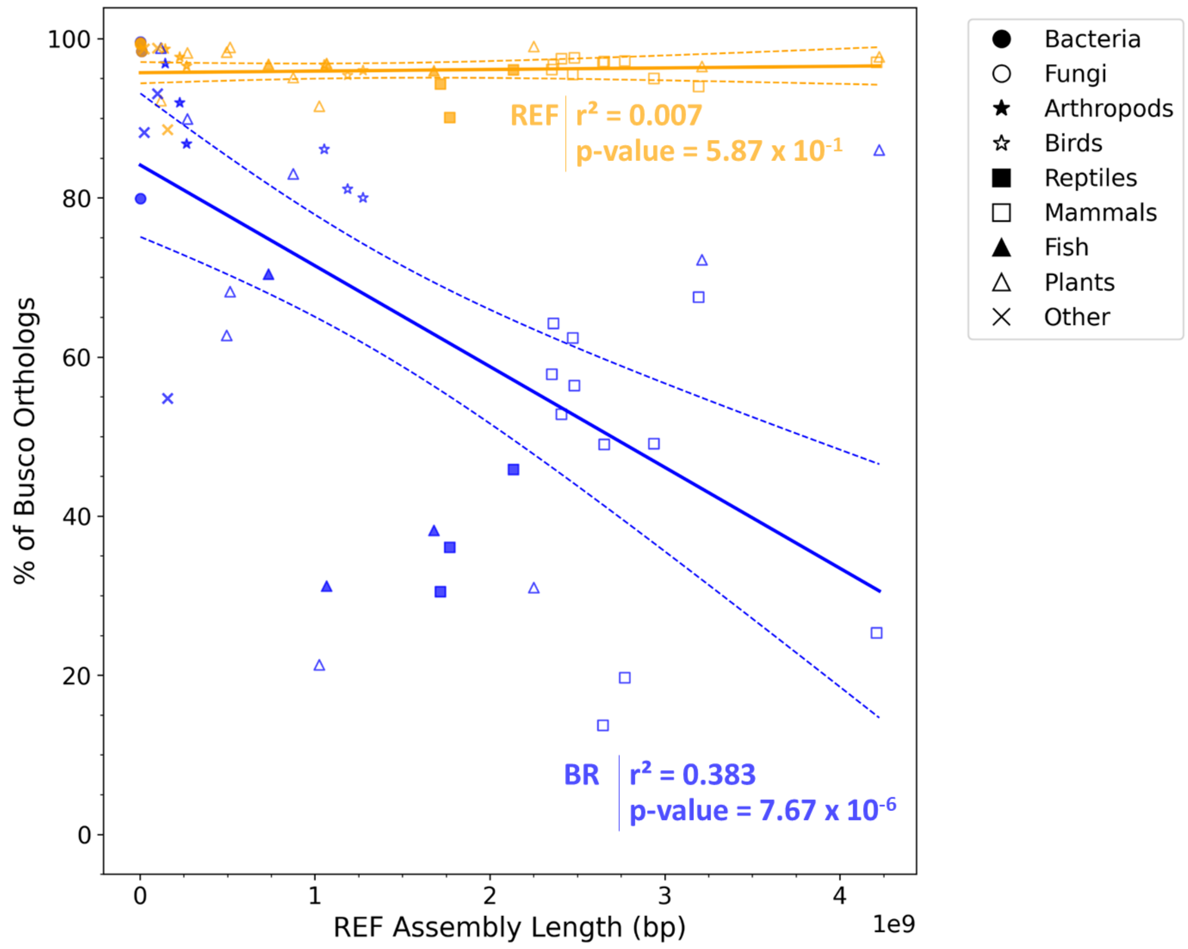

Figure S2. Completeness of DNA assemblies according to their length

The completeness of the REF and BR assemblies was assessed using sets of Benchmarking Universal Single-Copy Orthologs (Busco), based on evolutionary expectations of gene content. The fraction of the theoretical number of Busco orthologs found in assemblies is given as a function of their length. Linear regression r^2 and p-values are given, and confidence intervals (95%) are shown using dashed lines. Each point represents a species.

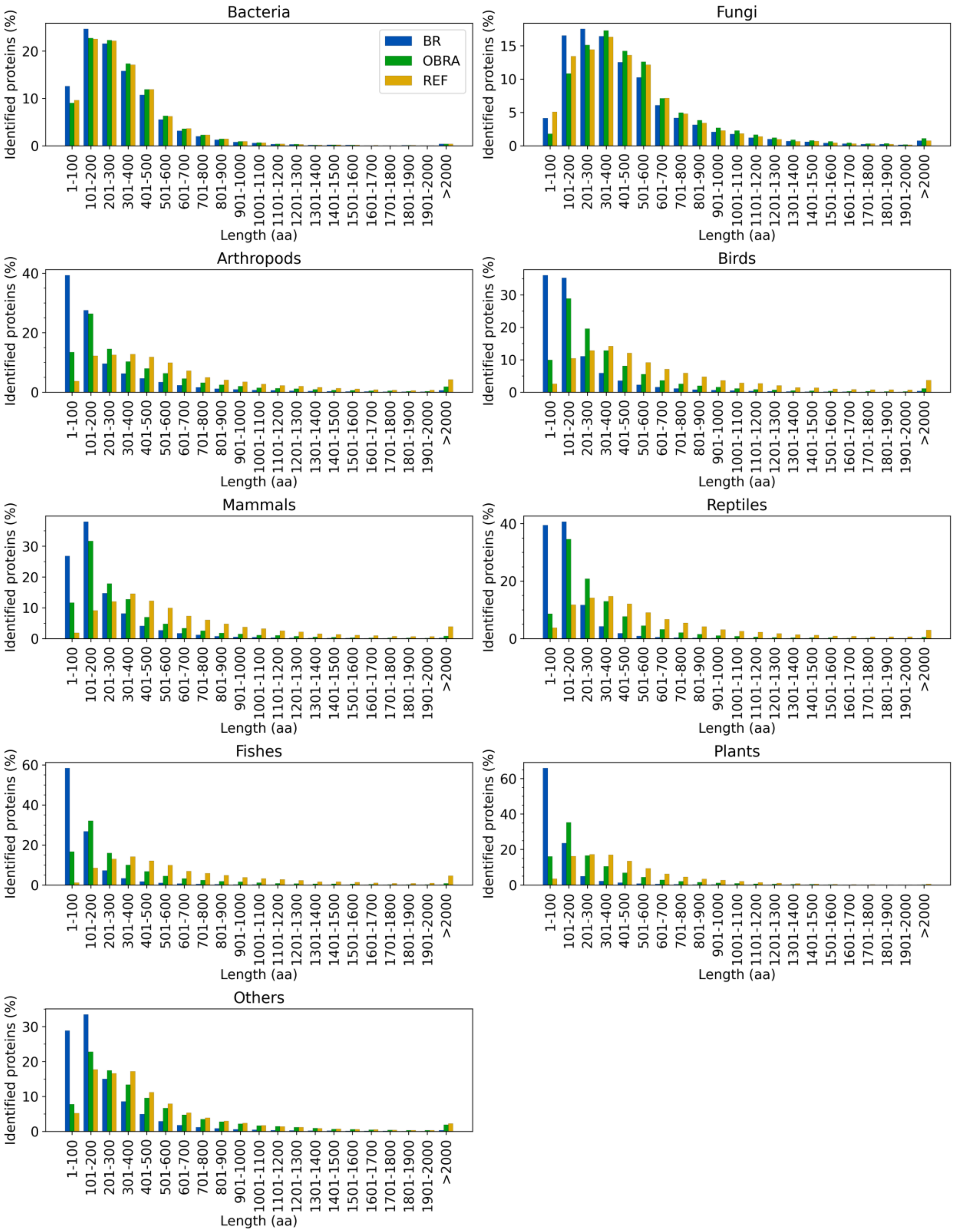

Figure S3. Size distribution of predicted proteins in the reference (REF) and Brownotate (BR and OBRA) annotations

The % of predicted proteins is given as a function of their size in 100 bp steps for the reference (REF, in yellow) and Brownotate (BR in blue, OBRA in green) annotations.

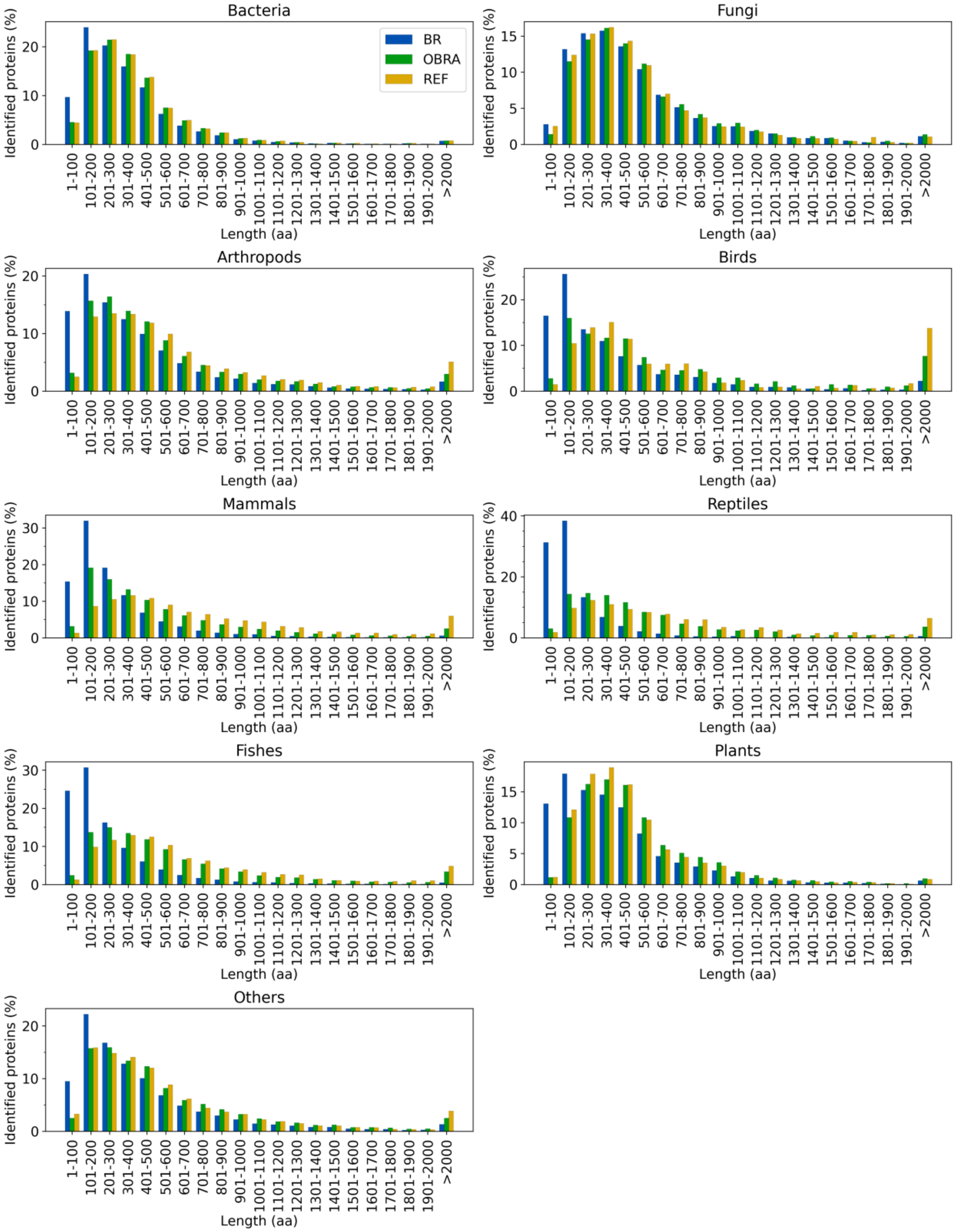

Figure S4. Size distribution of the proteins identified by MaxQuant when using the reference and Brownotate annotations

The % of predicted proteins is given as a function of their size in 100 bp steps for the reference (REF, in yellow) and Brownotate (BR in blue, OBRA in green) annotations.

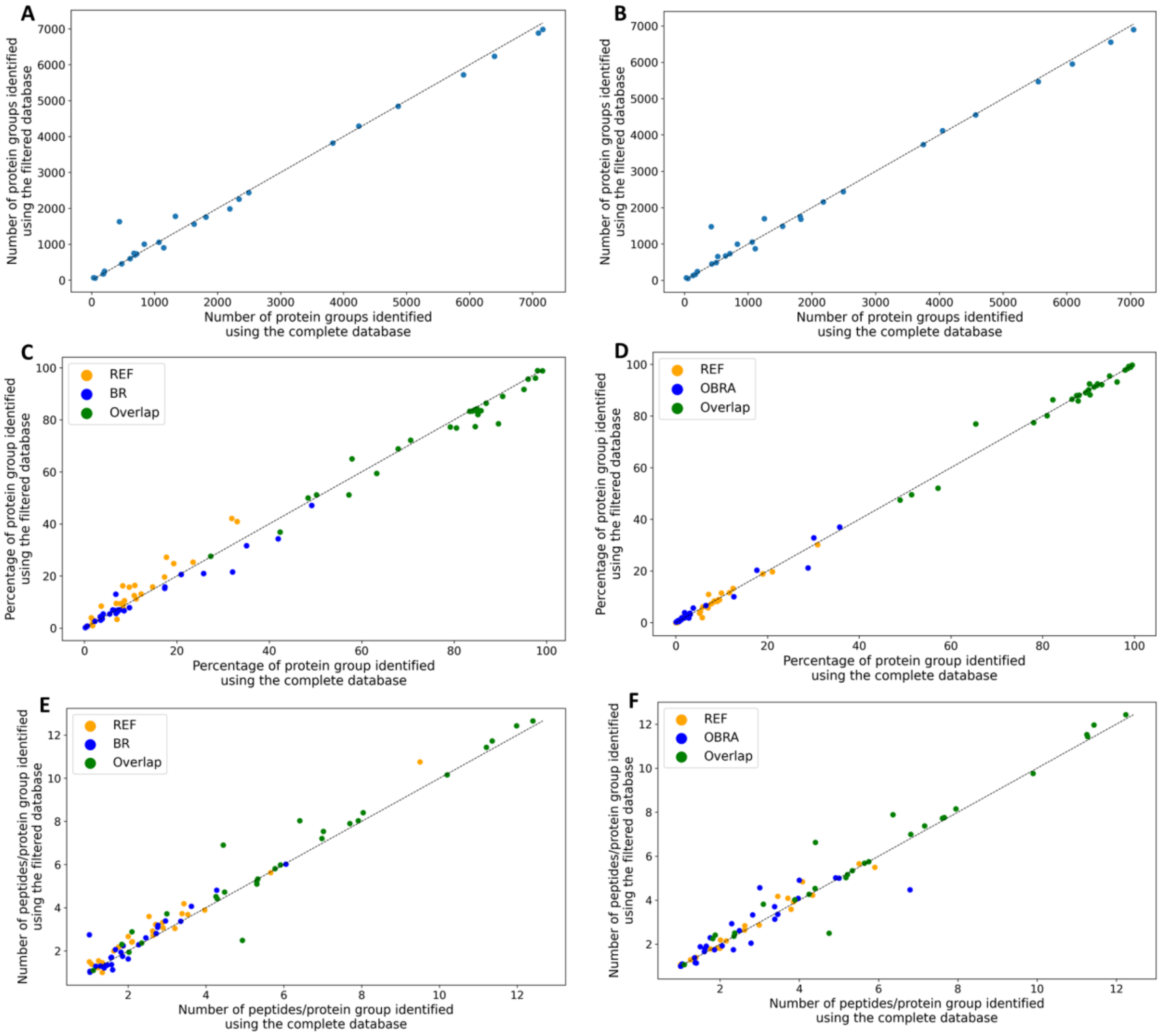

Figure S5. MaxQuant results obtained with or without discarding small proteins of less than 100 amino acids in protein sequence databases

The results obtained with complete protein sequence databases (i.e. containing all proteins including those of less than 100 amino acids) are plotted against those obtained with filtered databases (i.e. after discarding proteins of less than 100 amino acids). The dotted line indicates the theoretical case where results are identical regardless of the database used. **A** and **B.** Number of protein groups identified using REF versus BR (A) and using REF versus OBRA (B) databases. **C** and **D.** Percentage of protein groups identified solely from REF protein databases (yellow), solely from BR protein databases (C, in blue), solely from OBRA protein databases (D, in blue) or from both REF and BR or REF and OBRA databases (in green). **E** and **F.** Number of peptides per protein group for the proteins identified solely from REF protein databases (yellow), solely from BR protein databases (E, in blue), solely from OBRA protein databases (F, in blue) or from both REF and BR or REF and OBRA databases (in green). Exact values and dataset details are provided in Supplementary Table S9.
