# Supplementary material for: Brownotate, a Comprehensive Solution to Generate Protein Sequence Databases for Any Species
Source: Proteomics. 2026 Jan 6;26(5):13–26. doi: 10.1002/pmic.70094 (PMC13106930; doi:10.1002/pmic.70094)
Supplement: Supplementary file 2 — Supporting File 2: pmic70094‐sup‐0002‐Tables.pdf. [file PMIC-26--s007.pdf]

Brownotate, a comprehensive solution to generate protein sequence databases for any species

Adrien Brown[1,2], Alexandre Burel[1,2], Sarah Cianférani[1,2], Christine Carapito[1,2], Fabrice Bertile[1,2]

[1] Université de Strasbourg, CNRS, IPHC UMR7178, Laboratoire de Spectrométrie de Masse BioOrganique (LSMBO), 25 rue Becquerel, 67087 Strasbourg, France.

[2] Infrastructure Nationale de Protéomique ProFI – FR2048, 67087 Strasbourg, France.

Supplementary tables

Table S1. Reference sequencing datasets used to evaluate Brownotate performance

Table S2. Reference assemblies and annotations used to evaluate Brownotate performance

Table S3. Extrinsic sequences used to train the HMM models to generate protein sequence databases with Brownotate

Table S4. Proteomic datasets from PRIDE repository used to evaluate Brownotate performance

Table S5. PRIDE datasets and raw files used for MaxQuant searches

Table S6. Influence of the sequencing platform or strategy on the quality of Brownotate assembly

Table S7. Influence of the sequencing depth on the quality of Brownotate assembly

Table S8. Number of protein sequences and evaluation of redundancy in the reference and Brownotate annotations

Table S9. MaxQuant results obtained with or without discarding small proteins of less than 100 amino acids in protein sequence databases

Table S1. Reference sequencing datasets used to evaluate Brownotate performance

See corresponding Excel file.

Table S2. Reference assemblies and annotations used to evaluate Brownotate performance

Species	Accession	Strain	Assembly Date	Assembly level	Depth of sequencing	Assembly method	Annotation Date	Annotation provider	# Proteins
S. aureus **(Ba$_1$)**	GCF_000013425.1	NCTC 8325	Feb 13, 2006	Complete Genome	N/A	/	3 Aug 2016	NCBI RefSeq	2 767
L. brevis **(Ba$_2$)**	GCF_001676805.1	NPS-QW-145	Jun 27, 2016	Complete Genome	55x	Newbler v. v2.8; SSPACE Standard v. v3.0; GapFiller v. v1.10; CONTIGuator v. June 2011	25 Mar 2023	NCBI RefSeq	2 409
M. xanthus **(Ba$_3$)**	GCF_000012685.1	DK 1622	Jun 7, 2006	Complete Genome	N/A	/	22 Feb 2023	NCBI RefSeq	7 185
E. nidulans **(Fu$_1$)**	GCF_000149205.2	FGSC A4	Mar 1, 2012	Scaffold	N/A	/	24 Jan 2018	Broad Institute	9 556
S. cerevisiae **(Fu$_2$)**	GCF_000146045.2	S288C	Dec 17, 2014	Complete Genome	N/A	/	26 Jan 2024	SGD	6 016
F. oxysporum **(Fu$_3$)**	GCF_000271745.1	NRRL 32931	Feb 6, 2014	Scaffold	195x	ALLPATHS v. R36504	18 Apr 2022	Broad Institute	23 735
A. bisporus **(Fu$_4$)**	GCF_000300575.1	H97	Nov 30, 2012	Scaffold	8.5x	Arachne v. 20071016_modified	/	/	/
B. botryosum **(Fu$_5$)**	GCA_000697705.1	FD-172 SS1	Jun 05, 2014	Scaffold	31x	Newbler v. 2.5	/	/	/
R. toruloides **(Fu$_6$)**	GCF_000320785.1	NP11	Apr 01, 2013	Scaffold	200x	SOAPdenovo v. 1.04	/	/	/
D. melanogaster **(Ar$_1$)**	GCF_000001215.4	/	Aug 1, 2014	Chromosome	N/A	/	26 Dec 2023	FlyBase	30 717
A. gambiae **(Ar$_2$)**	GCF_000005575.2	PEST	Oct 16, 2006	Chromosome	N/A	VectorBase	3 Apr 2023	The International Consortium for the Sequencing of Anopheles Genome	14 102
A. mellifera **(Ar$_3$)**	GCF_003254395.2	DH4	Sep 10, 2018	Chromosome	192x	FALCON v. 0.5.0; Arcs v. 1.0.1; Links v. 1.8.5; BioNano Solve v. 3.1	13 Sep 2018	NCBI RefSeq	23 471
G. gallus **(Bi$_1$)**	GCF_016699485.2	/	Jan 19, 2021	Chromosome	102.01x	TrioCanu v. 1.8; purge_dups v. 1.0.0; Scaff 10x v. 4.1.0; Bionano solve v. 3.2.1_04122018; Salsa2 HiC v. 2.2; Arrow polishing and gap filling v. SMRTLink7.0.1; Freebayes v. 1.3.1; gEVAL manual curation v. 2020-07-22; VGP trio assembly pipeline v. 1.6	23 Dec 2021	NCBI RefSeq	68 683
C. caeruleus **(Bi$_2$)**	GCF_002901205.1	/	Jan 26, 2018	Scaffold	40x	Celera Assembler v. 7; IDBAUD v. MAY-2014	5 Feb 2018	NCBI RefSeq	31 326
S. demersus **(Bi$_3$)**	GCA_010077935.1	/	Jan 31, 2020	Scaffold	115x	Supernova v. 2.0.0	31 Jan 2020	BGI-Shenzhen	14 240
N. naja **(Re$_1$)**	GCA_009733165.1	/	Dec 11, 2019	Chromosome	60x	canu v. 1.6; hirise v. Dec-2018; bionano access v. 1.3; supernova v. 2.x	22 Jul 2021	Genentech Inc.,	25 817
P. vitticeps **(Re$_2$)**	GCF_900067755.1	/	Feb 28, 2017	Scaffold	N/A	/	7 Apr 2017	NCBI RefSeq	38 712
C. caretta **(Re$_3$)**	GCF_023653815.1	/	Jun 7, 2022	Chromosome	109x	Racon v. 1.4.13; Medaka v. 1.2.0; Pilon v. 1.23; Salsa v. 2.3; nf-core/hic v. 1.1.0; Juicer v. 1.6; Redbean v. 2.5; LongStitch v. 1.0.1	29 Jun 2022	NCBI RefSeq	54 596
C. sabaeus **(Ma$_1$)**	GCF_015252025.1	WHO RCB 10-87	Nov 6, 2020	Scaffold	75x	SuperNova v. 1.0	23 Nov 2020	NCBI RefSeq	61 846

P. cinereus (**Ma$_2$**)	GCF_002099425.1	/	Apr 18, 2017	Contig	57.3x	Falcon v. 0.3.0	24 Apr 2017	NCBI RefSeq	46 908
U. arctos (**Ma$_3$**)	GCF_023065955.1	/	Apr 20, 2022	Scaffold	32x	HiFiASM v. September-2021; Juicer v. October-2021	5 May 2022	NCBI RefSeq	51 919
C. lupus (**Ma$_4$**)	GCF_011100685.1	/	Mar 10, 2020	Chromosome	100x	FALCON v. 0.5.0	/	/	/
O. orca (**Ma$_5$**)	GCF_937001465.1	/	May 03, 2022	Chromosome	34x	various	/	/	/
S. suricatta (**Ma$_6$**)	GCF_006229205.1	/	Jul 03, 2019	Chromosome	50x	3-D DNA Pipeline v. AUGUST-2017	/	/	/
C. asiatica (**Ma$_7$**)	GCF_000296735.1	/	Sep 19, 2012	Scaffold	66x	allpaths v. R42316 HAPLOIDIFY=True	/	/	/
B. taurus (**Ma$_8$**)	GCF_002263795.3	/	Jul 01, 2023	Chromosome	N/A	Falcon v. FEB-2016	/	/	/
D. leucas (**Ma$_9$**)	GCF_002288925.2	/	Aug 29, 2019	Scaffold	117x	re-scaffolding v. Mar-2019	/	/	/
O. aries (**Ma$_{10}$**)	GCF_016772045.2	OAR_USU_ Benz2616	Jul 20, 2023	Chromosome	N/A	Canu v. 1.8; Nanopolish v. 0.12.5; Salsa v. 2.2; PB Jelly v. 15.8.24; Freebayes v. 1.3.1	/	/	/
P. tigris (**Ma$_{11}$**)	GCF_018350195.1	/	May 13, 2021	Chromosome	78x	NextDenovo v. 2.2-beta.0	/	/	/
P. promelas (**Fi$_1$**)	GCF_016745375.1	/	Jan 24, 2021	Scaffold	70x	CANU v. 1.8	19 Feb 2021	NCBI RefSeq	48 455
D. rerio (**Fi$_2$**)	GCF_000002035.6	Tuebingen	May 9, 2017	Chromosome	N/A	/	2 Jun 2017	NCBI RefSeq	52 829
O. latipes (**Fi$_3$**)	GCF_002234675.1	Hd-rR	Jul 27, 2017	Chromosome	N/A	FALCON v. MAY-2015	31 Jan 2018	NCBI RefSeq	44 766
C. sativa (**Pl$_1$**)	GCF_900626175.2	/	Feb 14, 2019	Chromosome	100x	/	13 May 2020	NCBI RefSeq	33 674
A. thaliana (**Pl$_2$**)	GCF_000001735.4	/	Mar 15, 2018	Chromosome	N/A	/	20 Oct 2022	TAIR and Araport	48 265
R. chinensis (**Pl$_3$**)	GCF_002994745.2	/	Jan 15, 2019	Chromosome	80x	TIL-R v. Rev: 523; CANU v. 1.6	23 Mar 2021	NCBI RefSeq	48 188
B. distachyon (**Pl$_4$**)	GCF_000005505.3	Bd21	Jan 24, 2018	Chromosome	9.43x	ARACHNE v. 20071016_modified	/	/	/
H. vulgare (**Pl$_5$**)	GCF_904849725.1	/	Apr 1, 2021	Chromosome	25x	Hi-Canu (commit r9818)	/	/	/
V. vinifera (**Pl$_6$**)	GCF_030704535.1	Pinot Noir 40024	Aug 9, 2023	Complete Genome	40x	hifiasm v. v.15	/	/	/
C. annuum (**Pl$_7$**)	GCF_002878395.1	UCD-10X-F1	Jan 30, 2018	Chromosome	56x	Supernova v. 1.1-Chili-Pepper	/	/	/
P. aphrodite (**Pl$_8$**)	GCA_003013225.1	/	Mar 21, 2018	Scaffold	155x	AllPaths v. Dec-2014	/	/	/
L. ruthenicum (**Pl$_9$**)	GCA_041430385.1	L31	Aug 23, 2024	Chromosome	31x	hifiasm v. 0.16.1	/	/	/
P. falciparum (**Ot$_1$**)	GCF_000002765.5	/	Apr 7, 2016	Complete Genome	100x	/	10 Sep 2020	Plasmodium falciparum Genome Sequencing Consortium	5 384
A. queenslandica (**Ot$_2$**)	GCF_000090795.2	/	May 28, 2010	Scaffold	8x	PHRAPATTACK v. Feb 2007	7 Jun 2021	NCBI RefSeq	23 542
C. elegans (**Ot$_3$**)	GCF_000002985.6	Bristol N2	Feb 7, 2013	Complete Genome	N/A	/	22 Nov 2023	WormBase	28 546

Listed are the REF assembly and annotation datasets and associated metrics found in NCBI databases, which were used to run Brownotate and assess its performance. The genome coverage column indicates the estimated base coverage across the assembly, it correspond to what we presented as sequencing depth in Table 1. Species are defined using abbreviations (between brackets) as reported in Table 1. Ba: bacteria; Fu: fungi; Ar: arthropods; Bi: birds; Re: reptiles; Ma: mammals; Fi: fish; Pl: plants; Ot: other taxonomic classes.

Table S3. Extrinsic sequences used to train the HMM models to generate protein sequence databases with Brownotate

Species	Shared group	Taxon from which were taken extrinsic sequences	Strain	Accession	Annotation provider	Annotation Date	# Proteins
S. aureus (**Ba_1**)	-	-	-	-	-	-	-
L. brevis (**Ba_2**)	-	-	-	-	-	-	-
M. xanthus (**Ba_3**)	-	-	-	-	-	-	-
E. nidulans (**Fu_1**)	Aspergillus (genus)	*Aspergillus luchuensis*	IFO 4308	GCF_016861625.1	Fermentation Microbiology, Education and Research Center for Fermentation Studies, Faculty of Agriculture, Kagoshima University	30 Jan 2021	12 662
S. cerevisiae (**Fu_2**)	Saccharomyces (genus)	*Saccharomyces paradoxus*	CBS432	GCF_002079055.1	SC	3 Apr 2023	5 538
F. oxysporum (**Fu_3**)	Fusarium (genus)	*Fusarium fujikuroi*	IMI 58289	GCF_900079805.1	HMGU-IBIS	8 Feb 2018	14 810
D. melanogaster (**Ar_1**)	Drosophila (genus)	*Drosophila simulans*	w501	GCF_016746395.2	NCBI RefSeq	28 Oct 2021	14 256
A. gambiae (**Ar_2**)	Cellia (subgenus)	*Anopheles stephensi* (Asian malaria mosquito)	Indian	GCF_013141755.1	NCBI RefSeq	3 Sept 2020	12 692
A. mellifera (**Ar_3**)	Apis (genus)	*Apis cerana* (Asiatic honeybee)	Korean	GCF_001442555.1	NCBI RefSeq	11 Apr 2019	10 719
G. gallus (**Bi_1**)	Phasianinae (subfamily)	*Phasianus colchicus* (Ring-necked pheasant)	-	GCF_004143745.1	NCBI RefSeq	14 Nov 2019	16 222
C. caeruleus (**Bi_2**)	Paridae (family)	*Parus major* (Great Tit)	-	GCF_001522545.3	NCBI RefSeq	8 Apr 2020	15 069
S. demersus (**Bi_3**)	Spheniscus (genus)	*Spheniscus magellanicus* (Magellanic penguin)	-	GCA_010076225.1	BGI-Shenzhen	31 Jan 2020	15 289
N. naja (**Re_1**)	Elapidae (family)	*Ophiophagus hannah* (king cobra)	-	GCA_000516915.1	Naturalis Biodiversity Center	11 Dec 2013	18 445
P. vitticeps (**Re_2**)	Iguania (suborder)	*Anolis carolinensis* (green anole)	-	GCF_000090745.1	NCBI RefSeq	26 May 2016	19 365
C. caretta (**Re_3**)	Cheloniidae (family)	*Chelonia mydas* (Green sea turtle)	-	GCF_015237465.2	NCBI RefSeq	2 Sept 2021	19 752
C. sabaeus (**Ma_1**)	Cercopithecinae (subfamily)	*Cercocebus atys* (sooty mangabey)	-	GCF_000955945.1	NCBI RefSeq	23 Mar 2015	20 614
P. cinereus (**Ma_2**)	Diprotodontia (order)	*Trichosurus vulpecula* (common brushtail)	-	GCF_011100635.1	NCBI RefSeq	8 Oct 2020	22 532
U. arctos (**Ma_3**)	Ursus (genus)	*Ursus americanus* (American black bear)	-	GCF_020975775.1	NCBI RefSeq	10 Jan 2022	20 389
P. promelas (**Fi_1**)	Cyprinoidei (suborder)	*Megalobrama amblycephala* (Wuchang bream)	-	GCF_018812025.1	NCBI RefSeq	6 May 2022	29 877
D. rerio (**Fi_2**)	Danioninae (subfamily)	*Danionella cerebrum*	-	GCA_007224835.1	Charite - Universitatsmedizin Berlin	22 Jul 2019	23 944
O. latipes (**Fi_3**)	Oryzias (genus)	*Oryzias melastigma* (Indian medaka)	-	GCF_002922805.2	NCBI RefSeq	24 Sep 2020	23 519
C. sativa (**Pl_1**)	Cannabaceae (family)	*Trema orientale*	-	GCA_002914845.1	Geurts lab	2 Feb 2018	35 846
A. thaliana (**Pl_2**)	Arabidopsis (genus)	*Arabidopsis lyrata*	-	GCF_000004255.2	NCBI RefSeq	2 May 2017	29 817

R. chinensis **(Pl$_3$)**	Rosoideae (subfamily)	*Argentina anserina* (silverweed cinquefoil)	-	GCF_933775445.1	NCBI RefSeq	2 Sep 2022	19 620
P. falciparum **(Ot$_1$)**	Plasmodium (genus)	*Plasmodium yoelii*	17X	GCF_900002385.2	WTSI	23 May 2020	6 037
A. queenslandica **(Ot$_2$)**	Porifera (phylum)	*Oopsacas minuta*	-	GCA_024704765.1	Spongex	16 Aug 2022	16 264
C. elegans **(Ot$_3$)**	Caenorhabditis (genus)	*Caenorhabditis briggsae*	AF16	GCF_000004555.2	The C.briggsae Sequencing Consortium	10 Dec 2021	21 922

Listed are the extrinsic sequences used to train the Hidden Markov Model (HMM) to generate protein sequence databases with Brownotate. Species are defined using abbreviations (between brackets) as reported in Table 1. Ba: bacteria; Fu: fungi; Ar: arthropods; Bi: birds; Re: reptiles; Ma: mammals; Fi: fish; Pl: plants; Ot: other taxonomic classes.

Table S4. Proteomic datasets from PRIDE repository used to evaluate Brownotate performance

Species	PRIDE accession	Publication (DOI)	Instrument	Acquisition method	Quality control	# of raw	Raw file size	Organism part	Experiment type
S. aureus (**Ba_1**)	PXD031708	10.1039/D2CC01259F, D (no access)	Q Exactive	NA	NA	8	11.1 Gb	Unknown	Shotgun proteomics
L. brevis (**Ba_2**)	PXD031809	Publication pending	Q Exactive HF-X	DDA	NA	28	24.1 Gb	Unknown	Shotgun proteomics
M. xanthus (**Ba_3**)	PXD031267	Publication pending	Exactive Plus	DDA	NA	24	31.3 Gb	Unknown	Shotgun proteomics
E. nidulans (**Fu_1**)	PXD015038	10.1074/mcp.ra119.001769	Orbitrap Fusion	DDA	NA	52*	19.8 Gb	Unknown	Shotgun proteomics
S. cerevisiae (**Fu_2**)	PXD031326	Publication pending	Q Exactive	NA	NA	10	10.0 Gb	Unknown	Shotgun proteomics
F. oxysporum (**Fu_3**)	PXD031137	Publication pending	Orbitrap Fusion Lumos	DDA	NA	8	11.3 Gb	Fungal cell	Shotgun proteomics
D. melanogaster (**Ar_1**)	PXD033608	Publication pending	Orbitrap Fusion Lumos	NA	NA	33	51.0 Gb	Testis	Shotgun proteomics<br>Affinity purification coupled with mass spectrometry proteomics
A. gambiae (**Ar_2**)	PXD016300	10.1002/pmic.201900400	Q Exactive	DDA	NA	15	15.6 Gb	Whole body	Shotgun proteomics
A. mellifera (**Ar_3**)	PXD032755	10.1016/J.JINSPHYS.2022.104397	Q Exactive HF	DDA	NA	48	35.5 Gb	Unknown	Shotgun proteomics
G. gallus (**Bi_1**)	PXD023871	10.3389/fphys.2021.658711	Q Exactive	DDA	NA	45	37.5 Gb	Pectoral muscle	Shotgun proteomics
C. caeruleus (**Bi_2**)	PXD009822	10.1038/s42003-018-0247-8	Q Exactive	NA	NA	572*	8.1 Gb	Egg	Shotgun proteomics
S. demersus (**Bi_3**)	PXD018096	10.1021/acsomega.0c04983	Q Exactive	NA	NA	2	1.8 Gb	Blood plasma	Shotgun proteomics
N. naja (**Re_1**)	PXD020497	Publication pending	Orbitrap Fusion	NA	NA	30	25.6 Gb	Venom	Shotgun proteomics
P. vitticeps (**Re_2**)	PXD011171	Publication pending	Orbitrap Fusion Lumos	DDA	NA	18	25.2 Gb	Skeletal muscle<br>Brain<br>Heart	Shotgun proteomics
C. caretta (**Re_3**)	PXD029569	10.1016/J.JPROT.2021.104433	Q Exactive	DDA	NA	9	7.4 Gb	Blood plasma	Shotgun proteomics
C. sabaeus (**Ma_1**)	PXD021297	10.1016/j.medntd.2022.100156	timsTOF Pro	DDA	NA	8	14.7 Gb	Cell culture	Shotgun proteomics
P. cinereus (**Ma_2**)	PXD024250	10.1002/pmic.202100067 (No access)	Q Exactive Plus	NA	NA	8	8.3 Gb	Sperm	Shotgun proteomics
U. arctos (**Ma_3**)	PXD004908	10.1186/s12983-019-0312-2	maXis 4G	DDA	Mean CV of peptide retention times for GAPDH (housekeeping protein: 3.3%. Median CV of LFQ values from all proteins across repeated injections of a reference sample: 25%	290*	7.4 Gb	Skeletal muscle	Shotgun proteomics
P. promelas (**Fi_1**)	PXD021155	10.1021/acs.est.0c05942 (No access)	Orbitrap Fusion Lumos	NA	NA	12	23.2 Gb	Whole body	Shotgun proteomics

D. rerio (**Fi_2**)	PXD027191	Publication pending	LTQ Orbitrap Velos	DDA	NA	28	12.1 Gb	Embryonic stem cell Neuron	Shotgun proteomics
O. latipes (**Fi_3**)	PXD022153	10.1074/mcp.ra120.002306	Q Exactive HF	NA	NA	15	11.2 Gb	Unknown	Shotgun proteomics
C. sativa (**Pl_1**)	PXD012969	10.1101/815837	Orbitrap Fusion	NA	NA	49*	11.3 Gb	Embryo	Shotgun proteomics
A. thaliana (**Pl_2**)	PXD040435	10.1073/PNAS.2211258120	Orbitrap Fusion Lumos	NA	NA	33	17.0 Gb	Whole body	Affinity purification coupled with mass spectrometry proteomics
R. chinensis (**Pl_3**)	PXD036672	10.3389/fpls.2022.1041141	Q Exactive Plus	DDA	NA	6	5.8 Gb	Abscission zone	Shotgun proteomics
P. falciparum (**Ot_1**)	PXD039646	10.1038/s41467-023-37890-2	timsTOF Pro	DDA	NA	12	87.6 Gb	Blood	Shotgun proteomics
A. queenslandica (**Ot_2**)	PXD027246	10.1242/JEB.242820	Orbitrap Fusion Lumos	DDA	NA	18	23.1 Gb	Whole body	Shotgun proteomics
C. elegans (**Ot_3**)	PXD038019	10.1016/J.JBC.2022.102753	Orbitrap Fusion	DDA	NA	32*	27.1 Gb	Unknown	Top-down proteomics

Listed are the proteomic datasets downloaded from PRIDE repository and used to evaluate the identification of proteins with MaxQuant software using either reference protein databases (see annotations in Table S2) or the protein sequence databases generated with Brownotate. * indicates when only part of the available raw files were used to run MaxQuant software. Arbitrarily retained files are indicated in Supplementary Table S5. Species are defined using abbreviations (between brackets) as reported in Table 1. Ba: bacteria; Fu: fungi; Ar: arthropods; Bi: birds; Re: reptiles; Ma: mammals; Fi: fish; Pl: plants; Ot: other taxonomic classes. NA indicates missing information.

Table S5. PRIDE datasets and raw files used for MaxQuant searches

See corresponding Excel file.

Table S6. Influence of the sequencing platform or strategy on the quality of Brownotate assembly

See corresponding Excel file.

Table S7. Influence of the sequencing depth on the quality of Brownotate assembly

See corresponding Excel file.

Table S8. Number of protein sequences and evaluation of redundancy in the reference and Brownotate annotations

Species	# Proteins			% proteins shorter than 100 residues			# Clusters "REF and/or BR"			# Clusters "REF and/or OBRA"		
	REF	BR	OBRA	REF	BR	OBRA	only REF	REF and BR	only BR	only REF	REF and OBRA	only OBRA
S. aureus **(Ba$_1$)**	2 767	2 661	2 626	17,78	12,21	12,26	369	2 361	269	196	2 534	45
L. brevis **(Ba$_2$)**	2 409	3 085	2 453	9,42	23,47	10,56	500	1 903	627	10	2 393	54
M. xanthus **(Ba$_3$)**	7 185	7 507	7 299	6,3	7,86	7,04	79	7 081	314	76	7 084	181
E. nidulans **(Fu$_1$)**	9 556	14 399	6 388	1,53	4,39	2,07	1657	7 131	6 749	3 207	5 581	445
S. cerevisiae **(Fu$_2$)**	6 016	5 710	4 619	5,44	4,34	1,41	539	4 812	299	930	4 421	28
F. oxysporum **(Fu$_3$)**	23 735	24 786	12 677	6,06	3,71	1,65	3781	11 963	11 046	3 550	12 194	230
D. melanogaster **(Ar$_1$)**	30 717	27 290	15 539	4,53	23,06	8,23	1694	11 756	9 799	1 873	11 577	2 381
A. gambiae **(Ar$_2$)**	14 102	38 308	12 500	5,16	44,46	6,65	2190	8 994	5 514	2 643	8 541	2 375
A. mellifera **(Ar$_3$)**	23 471	37 513	25 072	1,41	44,15	19,21	861	8 824	19 808	1 037	8 648	14 672
G. gallus **(Bi$_1$)**	68 683	42 803	16 859	1,38	18,98	4,95	4433	12 877	21 509	6 632	10 678	5 202
C. caeruleus **(Bi$_2$)**	31 326	44 801	31 059	2,07	39,09	15,21	3670	11 973	20 834	4 189	11 454	15 072
S. demersus **(Bi$_3$)**	14 240	95 025	59 237	8,38	40,98	7,92	2839	10 284	28 776	2 916	10 207	23 622
N. naja **(Re$_1$)**	25 817	222 748	32 921	12,58	38,87	10,39	9380	13 552	99 788	10 719	12 213	17 979
P. vitticeps **(Re$_2$)**	38 712	130 687	82 074	1,38	42,87	12,23	4853	13 918	68 677	5 080	13 691	49 026
C. caretta **(Re$_3$)**	54 596	148 429	141 558	1,09	35,02	5,48	4006	15 894	63 691	5 221	14 679	54 086
C. sabaeus **(Ma$_1$)**	61 846	75 315	63 423	2,05	26,58	11,95	6056	15 442	41 443	7 588	13 910	33433
P. cinereus **(Ma$_2$)**	46 908	66 153	49 656	1,66	27,9	12,81	4371	15 503	34 697	5 792	14 082	28 337
U. arctos **(Ma$_3$)**	51 919	46 776	44 687	1,56	23,24	8,32	5539	14 498	22 543	5 943	14 094	20 183
P. promelas **(Fi$_1$)**	48 455	153 895	91 247	1,12	47,88	16,39	3307	20 448	71 745	4 587	19 168	46 114
D. rerio **(Fi$_2$)**	52 829	297 967	102 759	1,25	67,95	19,47	4667	19 830	103 717	5 902	18 595	48 806
O. latipes **(Fi$_3$)**	44 766	132 088	62 484	0,85	46,1	10,51	3265	17 892	57 156	4 963	16 194	27 808
C. sativa **(Pl$_1$)**	33 674	717 286	179 637	2,01	76,01	19,34	2302	19 122	166 558	2 748	18 676	117 600
A. thaliana **(Pl$_2$)**	48 265	132 539	31 338	5,85	19,32	6,46	3049	22 355	99 646	3 161	22 243	6 222
R. chinensis **(Pl$_3$)**	48 188	591 129	109 042	1,87	62,29	12,12	2853	23 126	218 760	4 112	21 867	52 019
P. falciparum **(Ot$_1$)**	5 384	9 091	5 848	3,36	26,29	5,83	538	4 683	2 489	102	5 119	600
A. queenslandica **(Ot$_2$)**	23 542	115 259	37 999	1,64	32,85	8,71	4327	11 472	78 887	1 510	14 289	15 324
C. elegans **(Ot$_3$)**	28 546	31 805	15 518	8,18	12,56	5,35	4397	13 175	14 046	4 818	12 754	1 538

The number of predicted protein sequences in the REF, BR and OBRA annotations is given for the 27 species with the percentage of sequence shorter than 100 residues. Sequence similarity between the predicted BR or OBRA proteins and the REF proteins using cd-hit v4.8.1 to group into 'clusters' all the sequences sharing at least 90% sequence similarity over a minimum of 50% of the shortest sequence. The table shows the number of clusters containing only REF, BR or OBRA sequences, or both REF and BR or OBRA sequences. Species are defined using abbreviations (between brackets) as reported in Table 1. Ba: bacteria; Fu: fungi; Ar: arthropods; Bi: birds; Re: reptiles; Ma: mammals; Fi: fish; Pl: plants; Ot: other taxonomic classes.

Table S9. MaxQuant results obtained with or without discarding small proteins of less than 100 amino acids in protein sequence databases

See corresponding Excel file.
